# Case report: AVN of the femoral head five year follow-up of the combination of ipsilateral femoral neck and sub-trochanteric fracture

**DOI:** 10.1016/j.tcr.2016.03.005

**Published:** 2016-05-12

**Authors:** Wei Zhang, Feng Zhu, Hanqing Dong, Yaozeng Xu

**Affiliations:** Department of Orthopaedics, The First Affiliated Hospital of Soochow University, Suzhou 215600, China

**Keywords:** Ipsilateral fractures, Proximal femur, Femoral neck and sub-trochanteric, AVN

## Abstract

To our knowledge, the type of combination of ipsilateral femoral neck and sub-trochanteric fracture is rare. And the long term follow-up is seldom been reported. A 60 year old woman suffered from a traffic accident. We gave her the intramedullary nail treatment for the combination of ipsilateral femoral neck and sub-trochanteric fracture, and the fracture indeed cured after one year and there is no clue of necrosis of the femoral head, but after 5 years, there is an evidence of necrosis of the femoral head. Combination of ipsilateral femoral neck and sub-trochanteric fracture should be kept in mind. Patients with this unusual fracture should be kept under surveillance for longer than might be thought currently to be necessary for there is a possibility of necrosis of the femoral head, even a nondisplaced femoral neck fracture.

## Introduction

Femoral neck fractures and intertrochanteric fractures often occur in elderly patients. But concomitant ipsilateral fractures of the proximal femur are uncommon. Patients who have this severe intra- and extracapsular hip fractures are often seen in old people with osteoporosis following a fall, and most of the type which has been reported is concomitant ipsilateral femoral neck and intertrochanteric fracture. There is no other surgery report about combination of ipsilateral femoral neck and sub-trochanteric fracture. So we report this case and discuss how to deal with it more efficiently.

## Case presentation

**Fig. 1:**
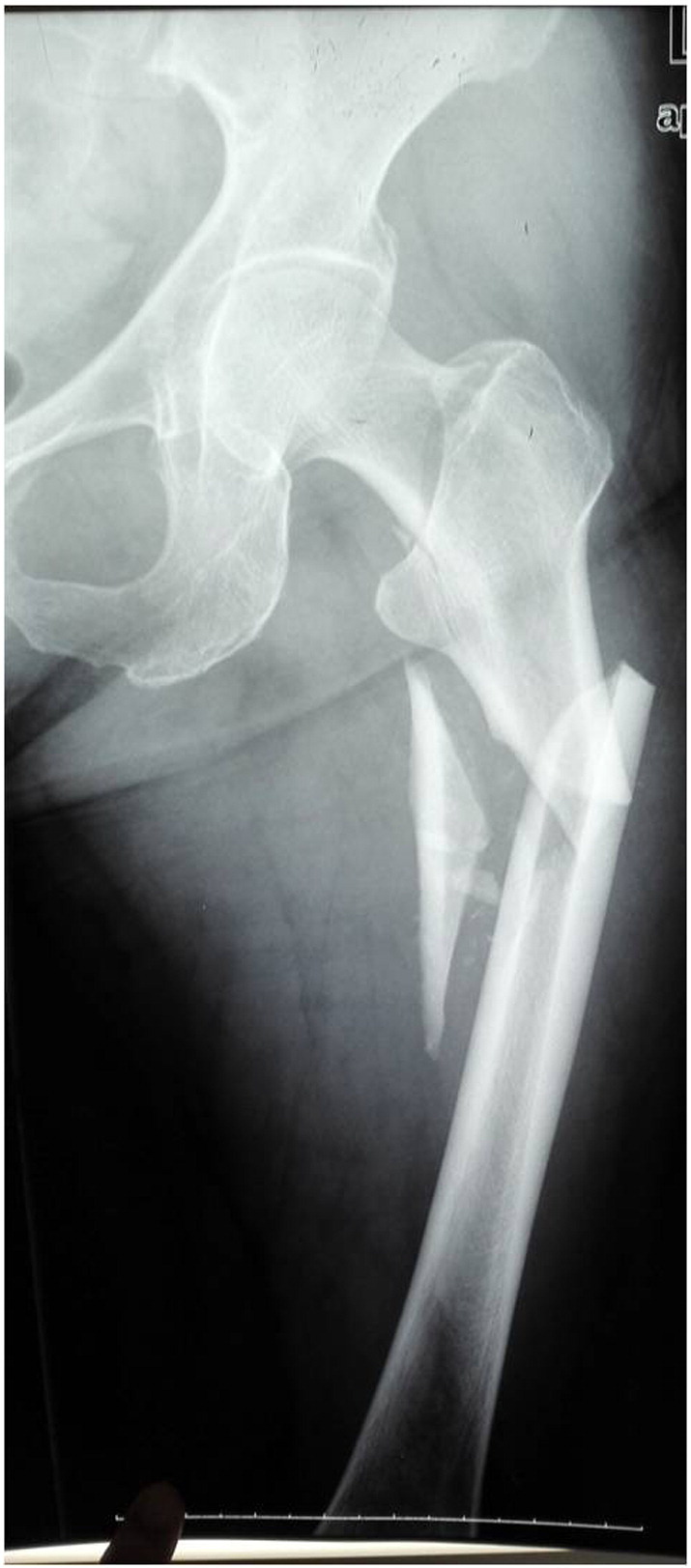
AP radiographic reveal a ipsilateral left femoral neck and sub-trochanteric fracture.

**Fig. 2:**
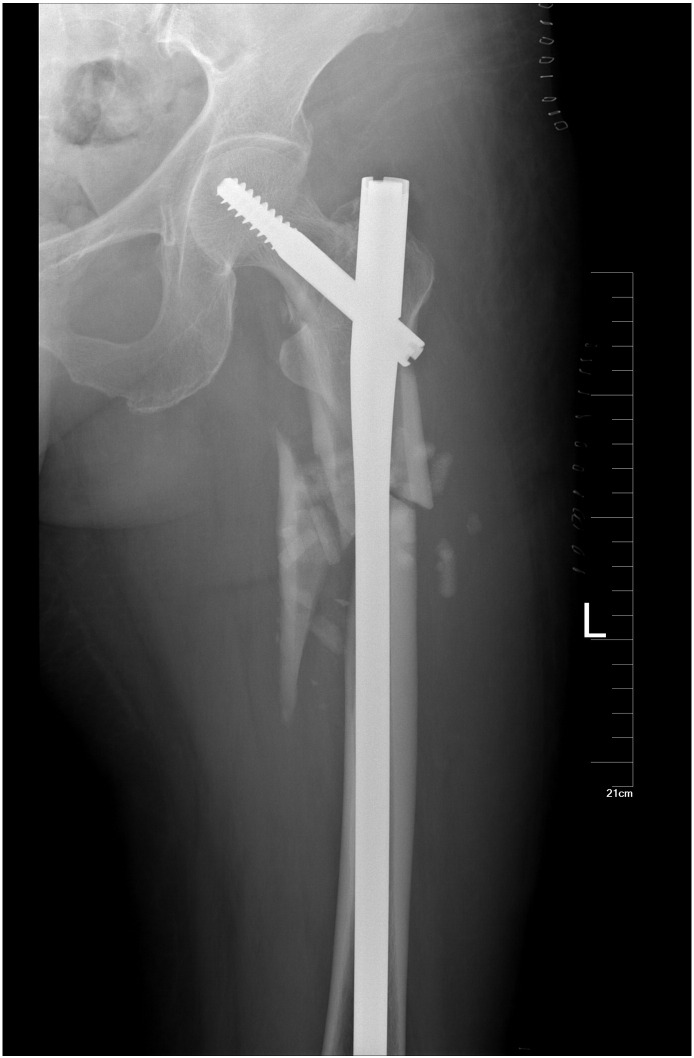
The fracture was fixed by a long Gamma3 nail.

**Fig. 3:**
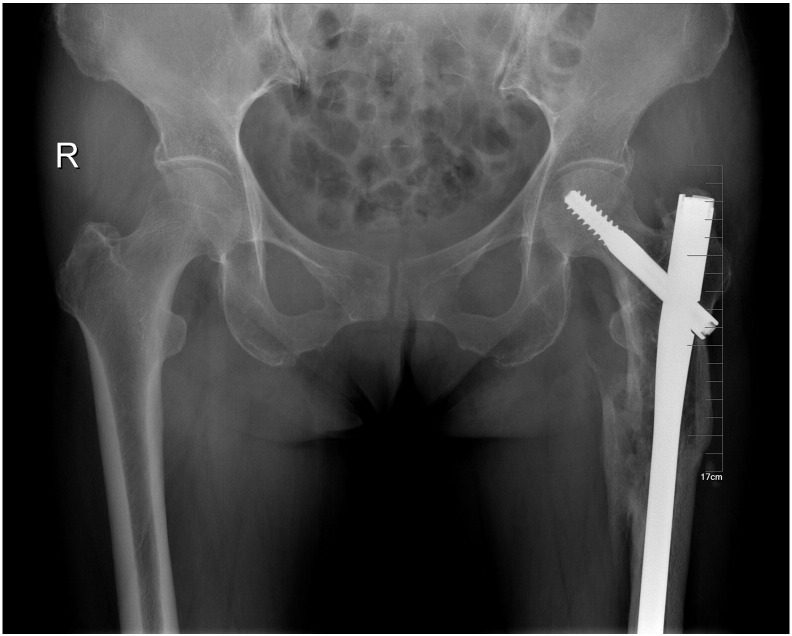
At 12-month follow-up, the fracture was union and has no evidence of avascular necrosis.

**Fig. 4:**
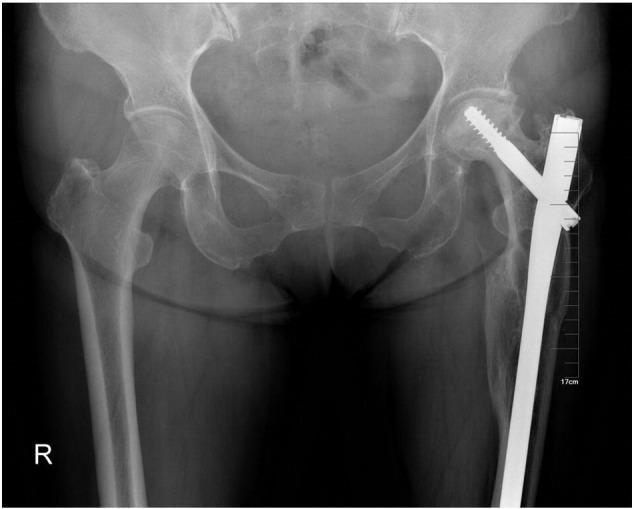
At 4 year follow-up, there was a evidence of the necrosis of the femoral head.

A 60-year-old female patient met with a traffic accident while walking. The patient had a coma and was taken to a nearby hospital for emergence treatment, then shifted to our hospital for further treatment after 2 weeks. On presentation, the patient was conscious, oriented and hemodynamically stable. The left lower limb was tracting, there was contusion over the hip. The right elbow was casting. The radiographs revealed a fracture of the right Ulna olecranon, a combination of ipsilateral left femoral neck and sub-trochanteric fracture. His neurovascular examination is normal. Evaluations are femoral neck fracture Garden II (nondisplaced) and sub-trochanteric fracture Seinsheimer IIIB (AO 32A1.1), AIS 14, ISS 27 (Fig. 1). Eventually the fracture was treated with a long Gamma3 nail (Fig. 2) with ORIF to the sub-trochanteric fracture, and the femoral neck fracture was treated with closed reduction. During fluoroscopy, care was taken to ensure that all the screw threads crossed the fracture lines and compression was obtained at the femoral neck region. After 12-month follow-up, the fracture was union and has no evidence of avascular necrosis (Fig. 3). But at 4-year follow-up, the patient began to complain of the pain. The X-ray showed an evidence of the necrosis of the femoral head (Fig. 4). One year later, she had a total hip arthroplasty for the severe pain.

## Discussion

Concomitant ipsilateral fractures in the proximal region of the femur are uncommon. There are 13 reports in the medical literature of ipsilateral fractures of the femoral neck and trochanteric region [1], [2], [3], [4], [5], [6], [7], [8], [9], [10], [11], [12], [13]. Of these, nine cases are reported in elderly osteoporotic patients following low energy fall [1], [2], [3], [4], [5], [6], [7], [9], [11]. One case was of a 54-year-old person caught in olive press [8]and the others were of patients with a motor vehicle accident [10], [12], [13]. And we also found one case of sub-trochanteric and neck fracture which was of a 56-year-old man with known autosomal-dominant osteoporosis [14]. Most of them had an operation and different implants were used, dynamic hip screw (DHS) with or without supplemental fixation, hemiarthroplasty with Parham bands, percutaneous compression plate, cancellous cannulated screws etc.(Table 1).The follow-up time from 3 to 58 months. Two in eight cases had the AVN [2], [4], [5], [8], [10], [11], [12], [13], one was 3 month postoperation and the other 12 month postoperation.

**Table 1:** List of all case report about intra- and extracapsular fractures of the proximal femur.

Author	Age/sex	Mechanism	Fracture type	Implant used	Follow-up	Outcome
An (1989)	NA	Low-energy	Intertrochanteric + neck	Hemiarthroplasty with Parham bands	NA	Good
Cohen (1999)	79/F	Low-energy	Pertrochanteric + subcapital	DHS	24 months	Ambulate cane, no AVN
Lawrence (1993)	NA	Low-energy	Intertrochanteric and subcapital	Pinning	NA	Patient died death not related to surgery
Kumar (2001)	83/F	Low energy	Intertrochanteric + subcapital	DHS + TSP + antirotation screw	12 months	Pain-free ambulation partial head collapse with AVN
Pemberton (1989)	73/F	Low energy	Subcapital + basicervical	DHS	30 months	Good, no AVN
Poulter (2007)	76/F	Low energy	Subcapital + intertrochanteric	PCCP	4 months	Good
Yuzo (2001)	89/F	Low energy	Neck + trochanter	Bipolar prosthesis	NA	Good
Sayegh (2005)	54/M	Olive press	Pertrochanteric + subcapital	DHS + cerclage wire	58 months	Good, no AVN, 2 cm short
Taniquchi (2013)	76/M	Low energy	Neck + reverse oblique	Prosthesis + LCP	6 monts	Union
Dhar (2008)	30/M	High-energy	T-shaped	DCP + lag screws	12 months	Good, no AVN
Perry (2008)	86/F	Low energy	Intertrochanteric + neck	DHS	3 months	Early AVN, failure, refused THR
Butt (2007)	30/M	High-energy	Neck + reverse oblique	DHS + antirotation screw	12 months	Good, no AVN
Neogi (2011)	28/M	High-energy	Neck + pertrochanteric	DCS + antirotation screw	28 months	Good, no AVN
Patrick Birmingham(2002)	56/M	Pathologic fracture	Sub-trochanteric + neck	Traction + cast	30 months	Mild limp, long distance with crane

In this case, the patient was cured in one year but had a necrosis at 4 year postoperation. There are little reports about such delayed necrosis. We think there might be three reasons for the necrosis, first, it is a high-energy traffic accident, the medial circumflex femoral artery might be injured; second, the operation time is too late, which caused the high pressure in the articular; and third the patient was a fat woman which means a heavy load to the hip. In our case a prosthetic replacement may be an option in the management of such injuries in elderly patients but it always means a long time hard work, we should prepare all revision tools. However, in a middle-aged, preserving the femoral head and at least putting off the time of the total hip arthroplasty (THA) should be the goal. A long IM nail is the most commonly used implant for the fixation of sub-trochanteric fractures, and cannulated screws are always used for the femoral neck fracture. But we use of a long Gamma3 nail to fix this combination injury for advantages below. First, long Gamma3 nail can be used for sub-trochanteric fracture, which is not suitable to DHS for the lateral cortex fracture. Second intramedullary fixation has less arm of force and matched the mechanics. Third Gamma3 nail has a lag screw which is easier to insert in young people than PFNA and can be used for compression of the femoral neck fracture [15]. Fourth the long Gamma3 nail has a distal target device to limit the dose of radiation [16]. Hence, in our cases we performed open reduction to the sub-trochanteric fracture and then close reduction to the femoral neck fracture under fluoroscopy and fixation with Gamma3 nail, one implant for two fractures. There is only one thing to be sure that the thread of lag screw across the femoral neck fracture line and Tip–apex distance (TAD) was also important.

## Conclusion

Concomitant ipsilateral intracapsular and extracapsular fractures are rare and the pattern which is ipsilateral femoral neck and sub-trochanteric fracture has not been reported. We must keep it in mind for diagnosis. In high-energy patients, femoral neck fracture can not only accompany an intertrochanteric fracture but can also accompany a sub-trochanteric fracture. Close examination of radiographs must be made to ensure that subtle fractures have never been overlooked. If doubt exists on initial radiographs further CT scan should be considered. We use Gamma3 nail to fix the sub-trochanteric fracture by open reduction and closed reduction to the femoral to minimise the damage to the artery. But in our case she had a necrosis at last, so patients with this unusual fracture should be kept under surveillance for longer than might be thought currently to be necessary, especially for patients receiving compensations.

## Transparency document

Transparency document.
